# Retraction: Perlecan and synaptophysin changes in denervated skeletal muscle

**DOI:** 10.4103/NRR.NRR-D-25-01216

**Published:** 2026-03-30

Following the publication of the article titled “Perlecan and synaptophysin changes in denervated skeletal muscle” (Ma et al., 2012; doi: 10.3969/j.issn.1673-5374.2012.17.002), concerns were raised regarding Figure 3.

In Figure 3, it appears that the second and fourth images in the “synaptophysin” column exhibit partial overlap. The second image seems to have been rotated counterclockwise by 90° and the resolution altered to create the fourth image. Given that these images are purported to represent different experimental conditions (the first week and third week of “time after denervation”), such manipulation raises serious questions about the authenticity of the data presented and the conclusions drawn in the article. This type of image manipulation is indicative of potential academic misconduct, which could mislead the scientific community.

In light of the concerns regarding multiple figure panels that question the reliability of the reported results and conclusions, the *Neural Regeneration Research* Editors have retracted this article.

YW did not agree with the retraction. KM, ZH, JM, LS, and HW could not be reached.
